# Assessing the Effects of Ginger Extract on Polyphenol Profiles and the Subsequent Impact on the Fecal Microbiota by Simulating Digestion and Fermentation In Vitro

**DOI:** 10.3390/nu12103194

**Published:** 2020-10-19

**Authors:** Jing Wang, Yong Chen, Xiaosong Hu, Fengqin Feng, Luyun Cai, Fang Chen

**Affiliations:** 1 Ningbo Research Institute, Zhejiang University, Ningbo 310027, China; wangjzju@zju.edu.cn (J.W.); fengfq@zju.edu.cn (F.F.); cailuyun@zju.edu.cn (L.C.); 2 College of Food Science and Nutritional Engineering, China Agricultural University, Beijing 100083, China; huxiaos@263.net; 3 College of Biosystems Engineering and Food Science, Zhejiang University, Hangzhou 310027, China; 15158157768@126.com; 4 School of Biological and Chemical Engineering, NingboTech University, Ningbo 310027, China; 5 College of Food Science and Biotechnology, Zhejiang Gongshang University, Hangzhou 310018, China

**Keywords:** in vitro fermentation, ginger extract, gut microbiota, 6-gingerol, short-chain fatty acids

## Abstract

The beneficial effects of ginger polyphenols have been extensively reported. However, their metabolic characteristics and health effects on gut microbiota are poor understood. The purpose of this study was to investigate the digestion stability of ginger polyphenols and their prebiotic effects on gut microbiota by simulating digestion and fermentation in vitro. Following simulated digestion in vitro, 85% of the polyphenols were still detectable, and the main polyphenol constituents identified in ginger extract are 6-, 8-, and 10-gingerols and 6-shogaol in the digestive fluids. After batch fermentation, the changes in microbial populations were measured by 16S rRNA gene Illumina MiSeq sequencing. In mixed-culture fermentation with fecal inoculate, digested ginger extract (GE) significantly modulated the fecal microbiota structure and promoted the growth of some beneficial bacterial populations, such as *Bifidobacterium* and *Enterococcus*. Furthermore, incubation with GE could elevate the levels of short-chain fatty acids (SCFAs) accompanied by a decrease in the pH value. Additionally, the quantitative PCR results showed that 6-gingerol (6G), as the main polyphenol in GE, increased the abundance of *Bifidobacterium* significantly. Therefore, 6G is expected to be a potential prebiotic that improves human health by promoting gut health.

## 1. Introduction

Ginger is the rhizome of *Zingiber officinale* Roscoe (*Zingiberaceae*) and is commonly consumed as spice or flavoring agent. Nowadays, ginger is widely used in making beverages and foods all over the world [1]. As a medicinal and edible plant, ginger has strong pharmacological activity and has been used as a Chinese herbal medicine for more than 2500 years. In recent years, the health-related applications of ginger have been widely studied, especially in various diseases, such as cancer [2], nausea and vomiting [3,4], gastrointestinal disease [5], osteoarthritis [6], and metabolic syndromes [7]. The nutritional and pharmacological activities of ginger mainly come from its bioactive polyphenols, especially the pungent principles gingerols and shogaols [8]. However, just a few studies have examined the digestion stability, bioavailability and metabolic characteristics of ginger and its bioactive phytochemicals [9,10]. The phenolics in ginger extract (GE) showed lower solubility in different pH buffers and remained stable in simulated gastric and intestinal fluids, indicating the suitability of these compounds during oral administration [11]. Additionally, similar stability of 6-shogaol, one of the major polyphenol compounds in ginger, was observed in simulated gastrointestinal fluids within 2 h [12]. As far as we know, most phenolic compounds are difficult to absorb in the small intestine, and then enter the colon to interact with the colonized gut microbiota [8]. The presence of these compounds in the colon leads to complex interactions between polyphenolic compounds and gut microbiota, including modulation of the gut microbiota by polyphenols and biotransformation of polyphenols by the gut microbiota [13]. Our previous research has shown that ginger can play an anti-obesity role by modulating the composition of gut microbiota [14]. However, the interactions between ginger polyphenols and the gut microbiota are still unclear.

In vitro simulated digestion and fermentation models are widely applied to predict bioavailability of foods or nutrients because these models are relatively inexpensive and without ethical concerns; moreover, the experimental conditions are controllable, the sampling is simple and the results are repeatable [15]. Several studies have focused on the potential applications of in vitro models. The biostability and catabolism of phenolic compounds in pomegranate have been reported using simulated gastrointestinal digestion and colonic fermentation models in vitro [16], and the importance of fruit seeds in the prevention of oxidative stress-related diseases has been evaluated using a similar approach [17]. In vitro approaches were also used to assess the polyphenol availability of açai and its subsequent health impact on the fecal microbiota [18]. Following in vitro digestion, fermentation of the nondigestible compounds in apple modulates bacterial populations in feces from obese mice with an increasing trend in the production of butyric acid [19]. However, to our knowledge, this study is the first to assess the effect of GE-containing phenolic compounds on changes in fecal bacteria in an in vitro model of fecal microbiota-mediated fermentation.

This study aimed to evaluate the stability of GE, which is rich in polyphenols and its beneficial effects on gut microbial using an in vitro model. Furthermore, the effect of 6G on promoting the amounts of specific genera was determined by quantitative real-time polymerase chain reaction (qPCR).

## 2. Materials and Methods

### 2.1. Plant Materials and Chemicals

After lyophilization, dried ginger (Laiwu, Shandong Province, China) was milled and stored in a moisture-controlled cabinet until use. The 6-, 8-, and 10-gingerols and 6-shogaol were purchased from Chromadex (Irvine, CA, USA). Unless otherwise noted, all chemicals were purchased from Sigma Chemical Co. (St. Louis, MO, USA).

### 2.2. Preparation of Ginger Extract

Dried ginger powder (200 g) was steeped in ethyl acetate (*w*:*v*, 1:5), stirred (4 °C) for 2 h and centrifuged (12,000× *g*, 4 °C) for 20 min. After extraction three times, the supernatants extracted continuously were combined, and the solvent was evaporated under reduced pressure at 40 °C using a rotary evaporator (IKA, Staufen, Germany). After lyophilization, the fractions were stored at −80 °C until use.

### 2.3. Simulated Digestion Model In Vitro

Simulated gastrointestinal and small-intestinal digestion of GE was carried out by a SPH-100D thermostat shaker (Shiping Instrument Corp., Shanghai, China) according to previous methods with minor modifications [20]. In brief, 0.5 g of dried GE was dissolved in 40 mL of Tris-maleate buffer (0.1 mol/L, pH 6.9) at 37 °C (water bath). For in vitro gastrointestinal digestion, the pH was adjusted to 2.0 using HCl (0.1 mol/L) solution; 16.5 μL of pepsin (≥250 units/mg; 160 mg/mL solution in 0.1 mol/L HCl) were then added, and the sample was incubated at 37 °C for 2 h for complete gastric digestion. In the simulated intestinal phase, 125 μL of pancreatin-bile extract (4 mg of pancreatin (350 FIP-U/g protease, 6000 FIP-U/g lipase, 7500 FIP-U/g amylase) and 25 mg of bile extract in 0.1 M NaHCO_3_) were added to the gastric mixture. After adjusting the pH to 7.5 using 0.1 M NaHCO3, digestion continued at 37 °C for another 2 h. Under the same conditions, culture media without GE was set as the control group. All experiments were carried out in the absence of light and oxygen by covering with foil and flushing with N_2_ for 10 min before digestion. Samples obtained from each digestion step were lyophilized and stored at −20 °C for analysis.

### 2.4. Simulated Fecal Fermentation In Vitro

Samples collected from the digestion described above were lyophilized for fecal fermentation in vitro. The culture medium was prepared according to a previously described method [21]. In brief, the sterile medium containing peptone (2 g/L), yeast extract (2 g/L), hemin (0.05 g/L), NaCl (0.1 g/L), MgSO_4_ 7H_2_O (0.01 g/L), CaCl_2_ 6H_2_O (0.01 g/L), bile salts (0.5 g/L), NaHCO_3_ (2 g/L), L-cysteine (0.5 g/L), K_2_HPO_4_ (0.04 g/L), KH_2_PO_4_ (0.04 g/L), Tween 80 (2 mL/L), vitamin K1 (10 mL/L), resazurin (1 mg/L) and distilled water. The medium was adjusted to pH 7.0 and continuously sparged with O_2_-free N_2_ overnight. The inoculum was prepared from fresh feces collected from male mice (C57BL/6 species) that were fed standard diets (D12450B; Research Diets) for 10 weeks and had not received antibiotics at any time. All experimental procedures were conducted with approval from the Biomedical Ethical Committee of Peking University (Beijing, China) with the approval number LA2018288. Freshly collected feces are immediately diluted with 10 mmol/L PBS (pH = 6.8) (*w/v*, 1:10). Next, the diluted feces were filtered with four layers of medical gauze. Then, the diluted feces were mixed with the culture medium (1:3) and the lyophilized fraction of the simulated digested GE from the process described above or the lyophilized fraction of digesta lacking GE (as a control). The mixture was distributed in disposable tubs (10 mL/tub/incubation time) and fermented at 37 °C in a LAI-3T anaerobic incubator (Longyue Instrument Corp., Shanghai, China) filled with gas mixture (H_2_ 5%, CO_2_ 10%, N_2_ 85%). All incubations were performed in triplicate. During the 24-h fermentation process, samples were collected at 0, 6, 12, and 24 h for analysis.

### 2.5. Identification and Quantification of 6-, 8-, and 10-Gingerols and 6-Shogaol at Different Time Points of Digestion and Fermentation In Vitro

Analyses were performed on a Waters Acquity UPLC I-class system (Waters, Milford, MA, USA) equipped with a dual pump, an autosampler and a Waters Xevo TQ-S MS detector (Waters, Milford, MA, USA) with an electrospray ionization (ESI) source. The data acquisition and analysis were controlled by ChemStation software (Waters, Milford, MA, USA). Chromatographic separation was carried out at 30 °C on a Waters Acquity UPLC BEH-C18 column (2.1 mm × 100 mm, 1.7 μm). The mobile phases consisted of 0.1% formic acid water (A) and acetonitrile (B) using a gradient elution of 40% to 0% (*v*/*v*) A at 0–0.5 min; 0% A at 0.5–1 min; 0–40% A at 1–2 min; and 40% A at 2–6 min. The flow rate was 0.2 mL/min and the injection volume was 2 μL. MS analysis conditions were as follows: drying gas (N_2_) flow rate, 9.0 L/min; drying gas temperature, 300 °C; nebulizer, 16 psi; capillary voltage, 4000 V; fragmentor voltage, 125 V. The standards and samples (digestion and fermentation) were analyzed in multiple reaction monitoring (MRM) mode using the ESI source by monitoring the [M + H]^+^. The content of 6-, 8-, smf 10-gingerols and 6-shogaol were expressed as μg/mg of digesta/fermented mixture. The retention time, quantitation ion and calibration curves for major polyphenolic compounds are shown in Table S1.

### 2.6. Estimation of the Total Polyphenol Content (TPC)

TPC was determined by the Folin–Ciocalteu method with 96-well plate microtitration [18]. In brief, 5 μL of the fermentation broth or standards were diluted with 145 μL of distilled water; the solution was then incubated for 5 min after 25 μL of Folin–Ciocalteu reagent was added. Then, 100 μL of 5% (*w*/*v*) Na_2_CO_3_ solution was added and the solution was then shaken for 25 min at room temperature. Finally, the absorbance was measured at 760 nm by Tecan Infinite M200 Pro microplate reader (Mannedorf, Switzerland). Different concentrations of gallic acid (0–500 mg/L) were used for making standard curves. TPC was expressed as gallic acid equivalent (GAE) per mg/g of freeze-dried GE.

### 2.7. DNA Extraction and MiSeq Sequencing

Genomic DNA was extracted using the TIANamp Stool DNA Kit (Tiangen, Beijing, China). Then, the DNA purity was then determined by 1% agarose gel electrophoresis. DNA samples were sequenced using primers 515F 5′-barcode-GTGCCAGCMGCCGCGG)-3′ and 907R 5′-CCGTCAATTCMTTTRAGTTT-3′, targeting the V3-V4 hypervariable regions of the 16S rRNA gene via MiSeq platform (IIIumina, San Diego, CA, USA) according to the manufacturer’s protocols at Shanghai MajorBio Bio Bio-Pharm Technology Co. Ltd. (Shanghai, China).

### 2.8. Processing and Bioinformatics Analysis of Sequencing Data

QIIME (version 1.17) was used for multiplexing and quality filtering of the raw fastq files. Operational taxonomic units (OTUs) were clustered with a 97% similarity cutoff using UPARSE (version 7.1). RDP Classifier (http://rdp.cme.msu.edu/) is employed to classify and analyze each of the 16S rRNA gene sequences in the Silva (SSU115) 16S rRNA database, with a confidence threshold of 70% [22]. To identify bacterial taxa with differences between different groups at the genus level, linear discriminant analysis effect size (LEfSe) was applied with linear discriminant analysis (LDA) score above 3.5 to measure the effect size of each abundant taxon and two filters (*p* < 0.05) [23]. PICRUSt was used to predict the function of microbial communities [24].

### 2.9. Analysis of pH Values and Short-Chain Fatty Acid (SCFA) Production

The pH values of GE and the control group were analyzed at 0, 6, 12, and 24 h during the fermentation process. The pH value of the supernatant of the fermentation broth after centrifugation (10,000× *g*, 10 min) was determined by a pH S-3B metera (Shanghai Leici Apparatus Corp., Shanghai, China).

The SCFA levels in the fermentation products were measured according to our previously described method [14]. The fermentation samples were centrifuged to obtain the supernatant fluid (0.8 mL). Then, 0.4 mL of 50% aqueous H_2_SO_4_ solution was added followed by vortex blending for 1 min. Subsequently, SCFA was extracted with 1.5 mL ethyl ether, shaken for 3 min, and centrifuged at 3000× *g* for 5 min. The residual water in the supernatant was removed with CaCl_2_. After centrifugation, 2 μL of the supernatant were injected into a gas chromatography (GC) system (Agilent 5975 GC) equipped with a polar GC column (ZB-FFAP, Phenomenex, CA, USA; 30 m × 0.32 mm × 0.25 μm). The solvent delay time was 3.5 min, the initial temperature was 90 °C for 2 min, then increased to 220 °C at the rate of 15 °C/min, and maintained at this temperature for 5 min. The temperatures of flame ionization detector and injection were both 175 °C. Carrier gas He flow rate was 1.0 mL/min. SIM mode and Agilent Mass Hunter WorkStation software (Agilent Technologies, Santa Clara, CA, USA) were used for data acquisition.

### 2.10. DNA Extraction and Quantitative Real-Time Polymerase Chain Reaction (qPCR)

Genomic DNA from the samples was extracted using the TIANamp Stool DNA Kit (Tiangen Biotech Co., Ltd., Beijing, China). Quantitative real-time PCR was performed using previously reported primers, and the sequences were as follows: Universal F: 5′-ACTCCTACGGGAGGCAGCAGT-3′, Universal R: 5′-GTATTACCGCGGCTGCTGGCAC-3′; *Bifidobacterium* F: 5′-TCGCGTCCGGTGTGAAAG-3′, *Bifidobacterium* R: 5′-CCACATCCAGCATCCAC-3′; *Enterococcus* F: 5′-TCCACGCCGTAAACGATGAG-3′, *Enterococcus* R: 5′-GACACGAGCTGACGACAACC-3′. Amplification and detection of DNA by qPCR were performed with a Light Cycler 480 real-time PCR instrument (Roche, Indianapolis, IN, USA). The reaction mixture consisted of 5 μL of SYBR Green I Master Mix (Roche, Indianapolis, IN, USA), 0.75 μL of each primer (10 mM), 2.5 μL of sterile water, and 1 μL of template DNA. A standard curve was prepared using continuous dilution of the purified and quantified PCR products generated by standard PCR and genomic DNA from mouse feces. Agarose gel electrophoresis (2% agarose) was used to ensure the correct prime-specific products were obtained. The concentration and quality of PCR products were measured by Nanodrop 2000 (Thermo Scientific, Wilmington, DE, USA). The amplification conditions were as follows: 95 °C for 5 min for initial denaturation, followed by 40 cycles of denaturation at 95 °C for 5 s, annealing at 60 °C for 30 s, extension at 72 °C for 30 s, and, finally, one cycle for melting curve analysis. The melting curve was checked after amplification to ensure that the melting temperature of the single product was consistent during amplification. Furthermore, PCR amplification products were randomly verified by gel electrophoresis (2% agarose). Every standard was run in triplicate. Gene copy numbers of individual samples were then calculated as standard curves derived from serial dilutions of reference strains. The results are reported as log gene copies per gram wet weight. All procedures were performed according to the Minimum Information for Publication of qPCR Experiments (MIQE) guidelines [25].

### 2.11. Statistical Analysis

The data are presented as the mean ± standard error of the mean [8]. For qPCR data regarding treatment, fermentation time and treatment × fermentation time, comparison of multiple samples was conducted by two-way ANOVA using SPSS 19 software (IMB Corp., Armonk, NY, USA). Other data were analyzed with simple one-way ANOVA with Duncan’s multiple range test, and *p* values < 0.05 were considered significant.

## 3. Results

### 3.1. Degradation of Polyphenols during Simulated Digestion and Fermentation

The TPC was assayed according to the Folin–Ciocalteu method. As shown in Table 1, the TPC of GE was reduced by 31% (from 884.9 ± 21.41 to 610.58 ± 12.45 GAE mg/g) after gastric digestiongastric digestion. However, a slight increase (23%) was observed after small-intestinal digestion. The stability and bioavailability of different polyphenols were also evaluated after simulated digestion in vitro. 6G was clearly present at the highest concentration (13.63 ± 0.49 μg/mg), followed by 8G (2.85 ± 0.30 μg/mg), 10G (1.33 ± 0.05 μg/mg), and 6S (1.85 ± 0.11 μg/mg), in the blank digesta. After simulated digestion, a significant decrease was observed in the quantity of 6G. The contents of other phenolics (8G, 10G, and 6S) also showed a downward trend, but it was not significant. During simulated digestion, the content of phenolic monomers showed a positive correlation with TPC.

Then, the digested GE was added to the fermentation medium mixed with mouse feces; the levels of individual phenolics and the TPC were further analyzed after collection of the fermentation supernatant samples at 0, 6, 12, and 24 h (Table 2). Following fermentation of GE with the fecal microbiota, a marked increase in TPC was observed for up to 12 h of fermentation, after which the TPC gradually decreased. The main phenolic compounds (6, 8, 10G, and 6S) showed a sharp decrease during the first 6 h and then showed a slight recovery in concentration for up to 12 h, subsequently decreasing within 24 h.

### 3.2. Changes in Bacterial Populations with In Vitro Batch Culture Fermentation

**Figure 1:**
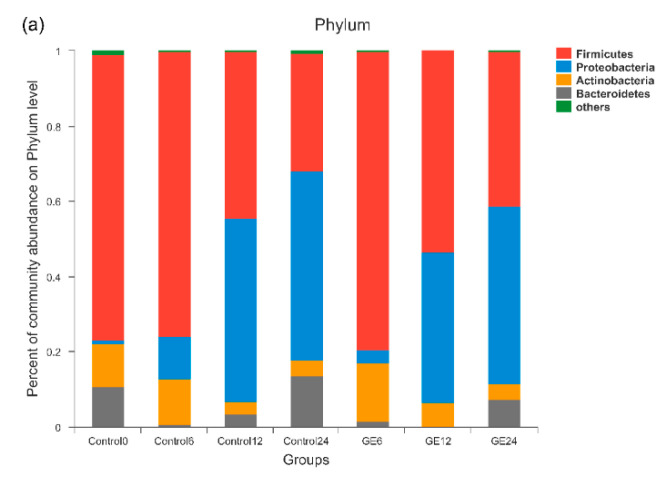

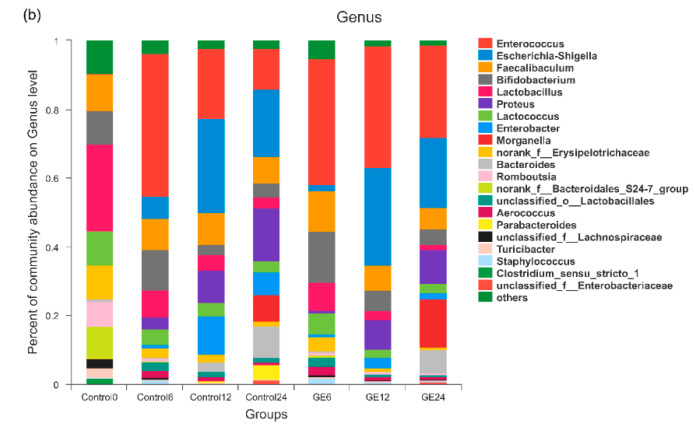
Relative abundance of the gut microbiota: (**a**) phylum level; and (**b**) genus level. Control0, Control6, Control12, and Control24 indicate fermentation of water at 0, 6, 12, and 24 h; GE6, GE12, and GE24 indicate fermentation of ginger extract (GE) at 6, 12, and 24 h.

To investigate the structural changes of the gut microbiota response to GE treatment, the effect of GE on changes in bacterial populations was analyzed at the phylum and genus levels after batch fermentation. As shown in Figure 1a, *Firmicutes*, *Bacteroidetes*, *Proteobacteria,* and *Actinobacteria* phyla were the dominant bacterial populations in the fecal samples. After 12 h of fermentation, the abundances of *Actinobacteria*, *Bacteroidetes,* and *Firmicutes* in both the control and GE groups declined notably, while the abundance of *Proteobacteria* increased significantly. Compared with the control group after fermentation for 12 h, GE greatly increased the abundance of *Actinobacteria* by 86.81% (*p* = 0.054) and *Firmicutes* by 20.94% (*p* = 0.047) but decreased the abundance of *Proteobacteria* by 17.9% (*p* = 0.074) and *Bacteroidetes* by 93.2% (*p* = 0.141). A similar trend was observed after 24 h of fermentation. The differences of microbiota structure in different groups at the genus level are shown in Figure 1b. After 12 h of fermentation, the abundances of the *Bifidobacterium* (*p* < 0.05) and *Enterococcus* (*p* < 0.01) were significantly higher in the GE group than in the control group, which also exhibited an increase after 24-h fermentation.

**Figure 2:**
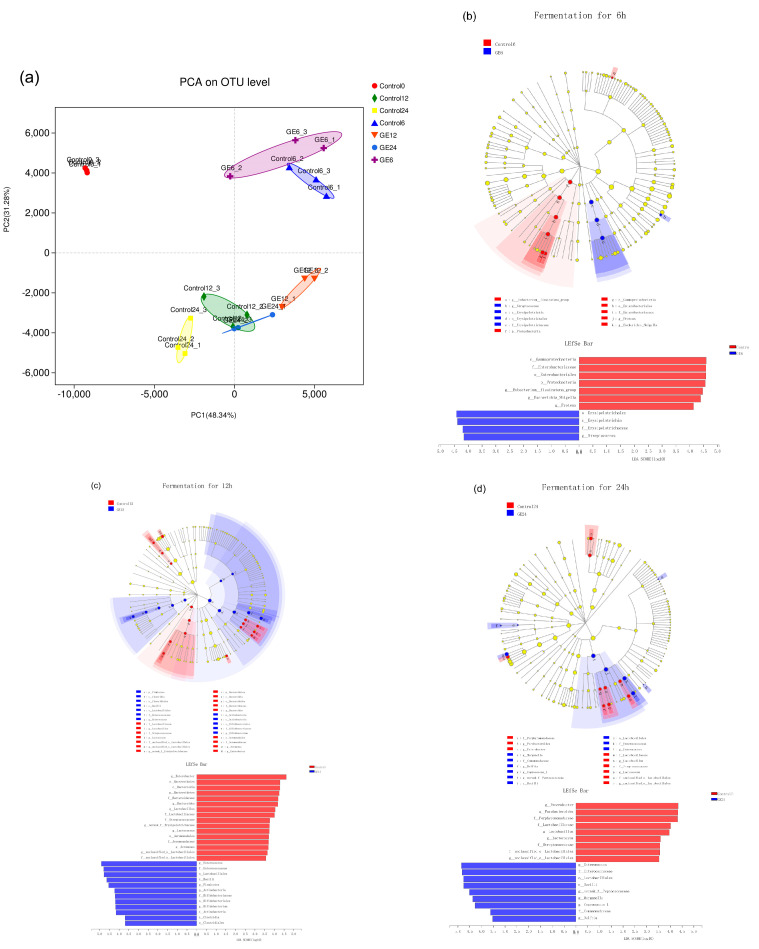
Comparison of the response of the gut microbiota to different treatments. (**a**) Principal component analysis of the gut microbiota at the operational taxonomic unit (OTU) level. Linear discriminant analysis (LDA) scores derived from LDA effect size (LEfSe) analysis of: 6-h fermentation (**b**); 12-h fermentation (**c**); and 24-h fermentation (**d**).

Principal component analysis (PCA) was used to reveal distinct clustering of fecal microbiota compositions from different samples. The results show that there were significant differences between the control group and other groups. Moreover, the GE fermentation groups were separated from the control groups (Figure 2a). Furthermore, the results of LEfSe analysis with 3.5 LDA score showed that the GE12 group was characterized by a high level of some bacterial strains (*Bifidobacterium*, *Bacilli*, and *Enterococcus)* compared with the control group. Consistent with the GE12 group, *Bacilli* and *Enterococcus* were identified as the predominant proteolytic taxa in the GE24 group (Figure 2b).

### 3.3. Effects of Ginger Extract on the pH Value and SCFA Production during In Vitro Fermentation

**Figure 3:**
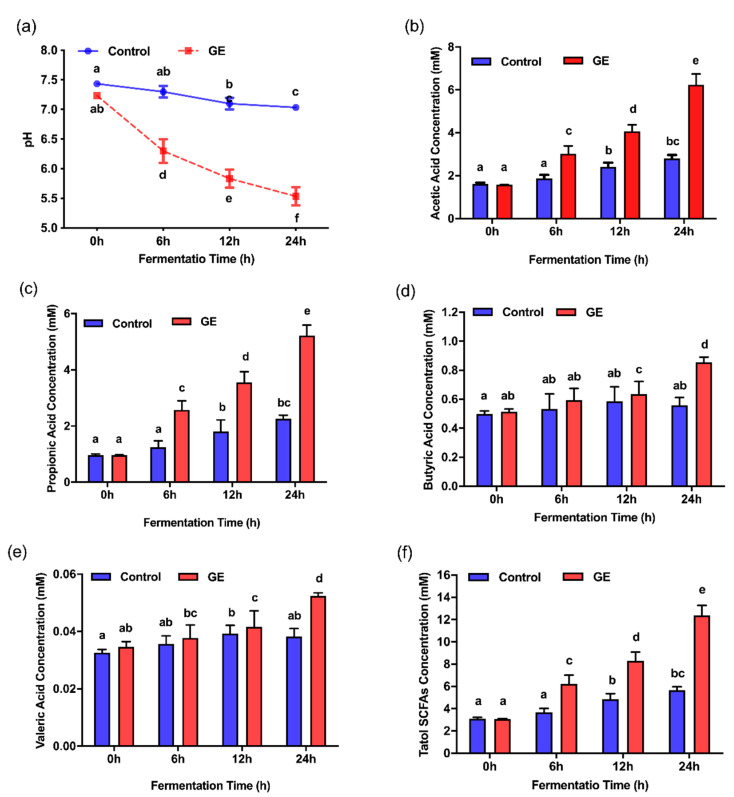
Concentrations of short-chain fatty acids (SCFAs) and pH values in predigested ginger extract (GE) or digestive juice (control) during in vitro fermentation: (**a**) pH; (**b**) acetic acid concentration; (**c**) propionic acid concentration; (**d**) butyric acid concentration; (**e**) valeric acid concentration; and (**f**) total SCFA concentration. All data are expressed as the mean ± SEM. Statistical analysis was performed using ANOVA. Means with different superscripts are considered to be significantly different (*p* < 0.05).

The pH values and SCFA concentrations of the fermentation supernatants at different time points during fermentation were examined. The pH value of the control group changed only slightly throughout the fermentation process. In contrast, the pH value of the GE treatment group decreased significantly with the extension of fermentation time compared to the control (*p* < 0.05), indicating high production of organic acids by fecal bacteria treated with GE (Figure 3a). We further evaluated SCFA production after 6, 12, and 24 h of incubation. In the fermentation supernatants, the concentrations of total SCFAs and several major individual SCFAs of the GE group were significantly higher than those of the control group at each time point (*p* < 0.05) (Figure 3b–f). After 24-h colonic fermentation, the levels of total SCFAs showed a significant increase from 3.09 ± 0.03 mM (0 h) to 12.36 ± 0.91 mM (24 h) in the GE treatment group.

### 3.4. Effect of 6G on the Specific Microbial Population during In Vitro Fermentation

To further understand the effects of 6G, the main polyphenol in GE, on the specific microbiota during in vitro fermentation, quantitative PCR was used to determine the abundances of total bacteria, *Enterococcus,* and *Bifidobacterium* following batch fermentation of fecal samples for 0, 6, 12, and 24 h. As shown in Table 3, the copy numbers of *Bifidobacterium* and *Enterococcus* decreased with extension of fermentation time, but the abundance of *Bifidobacterium* in the 6G fermentation group was higher than that in the control group during the entire fermentation period (*p* < 0.001). Additionally, no significant differences in total bacterial copies were observed between the 6G and control groups throughout the fermentation process.

## 4. Discussion

The interactions between polyphenols and the gut microbiota have received much attention due to the proposed health benefits. Polyphenols can balance the gut microbiota, resulting in specific changes in beneficial microbes, thus conferring benefits to host health [13]. GE, which contains many bioactive phenolics, such as shogaols and gingerols, exhibits several potential health benefits in both animal models and patients [26,27,28]. As the metabolism of polyphenols is currently being elucidated, it is important to understand how the process of gastrointestinal digestion or colonic fermentation affects ginger polyphenol bioaccessibility and the possible beneficial effects of these compounds in modulating the gut microbiota. Because of the practical, economic, and ethical limitations of in vivo intervention trials, in vitro colonic fermentation simulation with digestion models is considered to be a good approach for studying the metabolism and bioavailability of foods rich in polyphenols. Several in vitro digestion models combined with simulated intestinal fermentation models using human or mouse fecal inoculation have been used as valuable alternatives to in vivo studies [19,29,30,31]. However, since in vitro simulations cannot be exactly the same as in vivo conditions, verification through animal and/or human studies remains imperative.

First, our work demonstrated that approximately 15% of the TPC were destroyed, with considerable biotransformation and degradation observed during simulated gastric and intestinal digestion. Although the content of 6G decreased significantly during simulated digestion, 6G and other polyphenols were still detected in the digestive fluid. These results suggest that polyphenols present in GE are not fully degraded during the digestion process, and a significant percentage of these compounds reach the colon. Furthermore, it is well known that polyphenols are mainly metabolized by the gut microbiota in the colon. The microbial activities may contribute to catabolism into relatively small metabolites, such as SCFAs [32]. Moreover, these metabolites in turn regulate the bacterial composition in the gut [33]. In the simulated intestinal environment, the GE phenolics were shown to be further degraded by fecal bacteria, as the concentrations of gingerols and 6-shogaol decreased after fecal fermentation. Additionally, these phenolics modulated the bacterial composition with a resultant increase in SCFA synthesis. The two-way interaction between polyphenols compounds and the gut microbiota, including modulation of the gut microbiota with polyphenols and biotransformation of polyphenols by the polyphenols, which point to a new pathway in the prevention and treatment of many disease that threaten human health [13,34,35,36]. To date, many bioactive phenolics, such as açai [18], red wine [37], and berry [38], have demonstrated their health effects by batch colonic fermentation in vitro.

The potential prebiotic effect of GE on fecal bacteria was mainly reflected in the promotion of the growth of *Bifidobacterium* and *Enterococcus*. Similarly, increased abundances of *Bifidobacterium* and *Enterococcus* were observed with the addition of nondigestible compounds from apple in batch culture fermentation with feces from diet-induced obese mice [19]. Similarly, the abundance of *Bifidobacterium* and *Lactobacillus–Enterococcus* was observed to increase significantly after anthocyanins treatment in an in vitro human gut model [39]. *Enterococcus* has been reported to have beneficial effects in regulating inflammation and activating the immune system [40,41]. Moreover, members of the genus *Bifidobacterium* are known to be beneficial probiotic bacteria, and the growth pattern of *Bifidobacterium* was consistent with the observed SCFA production. SCFAs contribute to acidification of the batch fermentation environment, which is consistent with the decrease in pH value. Therefore, the acidic colon environment can contribute to the health of the host by promoting the proliferation of beneficial bacteria and inhibiting the growth of pathogens [42]. As expected, the acetic acid, propionate, butyrate, and total SCFA concentrations were all higher in the supernatants from the fermentation broths with the digested GE, which is related to the basal nutrient medium utilization by the increased SCFA-producing bacteria caused by GE. These processes mediated by the microbiota may play a key role in regulating the health-promoting effects and biological activities of ginger phenolics. Moreover, because 6G was clearly present at the highest concentration in the digesta before fermentation, the amounts of total bacteria, *Bifidobacterium,* and *Enterococcus* were further analyzed to evaluate the potential prebiotic effect of 6G. The results show that no significant difference in total bacterial copy numbers was found between the 6G group and the control group, while the copy numbers of *Bifidobacterium* were higher in the 6G group throughout the fermentation period, which is consistent with the potential positive effects of 6G on the gut microbiota. These results are preliminary, and further in vivo studies are needed to determine whether these potential prebiotic effects have health benefits for humans.

## 5. Conclusions

Our results reveal that GE could pass through the simulated digestion process without being fully degraded and could reach the colon. In the following simulated colonic environment model in vitro, a two-way interaction between GE and the gut microbiota showed that GE was degraded by fecal bacteria, which in turn modulated the composition of gut microbiota and promoted the growth of beneficial bacterial populations, such as *Bifidobacterium* and *Enterococcus*. Furthermore, incubation with GE could elevate the levels of SCFAs accompanied by a decrease in the pH value. Moreover, 6G, as the main polyphenol in GE, showed a potential prebiotic effect on the promotion of *Bifidobacterium* growth. Future research, which should include animal models and human cohort, is needed to confirm the mechanisms of interactions between 6G and gut microflora and its metabolites, as well as 6G metabolites. This may provide a new therapeutic application for 6G to exert health benefits through gut microbiota modulation.

## Figures and Tables

**Table 1:** Total polyphenolic content (TPC) and major polyphenolic compounds of ginger extract (GE) under simulated gastric and intestinal digestion. All data are expressed as the mean ± SEM. Statistical analysis was performed using ANOVA.

Treatments	TPC (GAE mg/g)	Polyphenolic Compounds (μg/mg of Digesta)			
		6G	8G	10G	6S
Blank	884.89 ± 21.41 ^a^	13.63 ± 0.49 ^a^	2.85 ± 0.30 ^a^	1.33 ± 0.05 ^a^	1.85 ± 0.11 ^a^
Gastric	610.58 ± 12.45 ^c^	9.02 ± 0.51 ^b^	2.91 ± 0.35 ^a^	1.26 ± 0.14 ^a^	1.75 ± 0.20 ^a^
Small intestine	751.03 ± 31.39 ^b^	7.09 ± 0.53 ^c^	2.80 ± 0.19 ^a^	1.05 ± 0.00 ^a^	1.68 ± 0.10 ^a^

Means with different superscripts are considered to be significantly different (*p* < 0.05). GAE, gallic acid equivalent; 6G, 6-gingerol; 8G, 8-gingerol, 10G, 10-gingerol; 6S, 6-shogaol.

**Table 2:** Total polyphenolic content (TPC) and major polyphenolic compounds of digested ginger extract (GE) under simulated fermentation. All data are expressed as the mean ± SEM. Statistical analysis was performed using ANOVA.

Incubation Period (h)	TPC (GAE mg/g)	Polyphenolic Compounds (μg/mg of Fermented Mixture)			
		6G	8G	10G	6S
0	523.86 ± 13.57 ^b^	4.81 ± 0.65 ^a^	1.36 ± 0.20 ^a^	1.10 ± 0.02 ^b^	0.86 ± 0.22 ^a^
6	542.25 ± 25.41 ^b^	1.43 ± 0.1 ^bc^	0.17 ± 0.01 ^b^	0.86 ± 0.00 ^c^	0.30 ± 0.00 ^b^
12	691.81 ± 21.35 ^a^	2.45 ± 0.68 ^b^	1.42 ± 0.36 ^a^	1.27 ± 0.09 ^a^	0.54 ± 0.10 ^ab^
24	432.64 ± 21.89 ^c^	0.50 ± 0.02 ^c^	0.17 ± 0.00 ^b^	0.86 ± 0.00 ^c^	0.30 ± 0.00 ^b^

Means with different superscripts are considered to be significantly different (*p* < 0.05). GAE, gallic acid equivalent; 6G, 6-gingerol; 8G, 8-gingerol, 10G, 10-gingerol; 6S, 6-shogaol.

**Table 3:** Copy numbers (log_10_ copies/g) of total bacteria, *Bifidobacterium,* and *Enterococcus* in fermentation broths under the normal or 6G treatment at different fermentation time points. All data are expressed as the mean ± SEM.

Bacteria	Group	Fermentation Time (h)				*p* Value		
		0	6	12	24	Treatment	Fermentation Time	Treatment × Fermentation Time
**Total bacteria**	Control	9.83 ± 0.63	9.70 ± 0.30	9.95 ± 0.20	10.10 ± 0.50	0.741	0.409	0.559
	6G	9.81 ± 0.49	9.78 ± 0.49	10.25 ± 0.16	9.87 ± 0.60			
**Bifidobacterium**	Control	4.55 ± 0.45	3.90 ± 0.16	3.75 ± 0.99	3.31 ± 0.22	<0.001	0.015	0.606
	6G	5.42 ± 0.33	5.15 ± 0.43	4.99 ± 0.55	4.79 ± 0.66			
**Enterococcus**	Control	7.33 ± 0.55	7.20 ± 0.27	6.43 ± 0.37	6.37 ± 0.56	0.425	0.003	0.71
	6G	7.47 ± 0.47	7.38 ± 0.69	6.96 ± 0.63	6.18 ± 0.70			

## Supplementary Materials

The following are available online at https://www.mdpi.com/2072-6643/12/10/3194/s1, Table S1: Retention time, quantitation ion, and calibration curves for major polyphenolic compounds of ginger.

## References

[1] Kou X., Wang X., Ji R., Liu L., Qiao Y., Lou Z., Ma C., Li S., Wang H., Ho C. (2018). Occurrence, biological activity and metabolism of 6-shogaol. Food Funct..

[2] Saxena R., Rida P.C.G., Kucuk O., Aneja R. (2016). Ginger augmented chemotherapy: A novel multitarget nontoxic approach for cancer management. Mol. Nutr. Food Res..

[3] Palatty P.L., Haniadka R., Valder B., Arora R., Baliga M.S. (2013). Ginger in the prevention of nausea and vomiting: A review. Crit. Rev. Food Sci..

[4] Terry R., Posadzki P., Watson L.K., Ernst E. (2011). The use of ginger (*Zingiber officinale*) for the treatment of pain: A systematic review of clinical trials. Pain Med..

[5] Haniadka R., Saldanha E., Sunita V., Palatty P.L., Fayad R., Baliga M.S. (2013). A review of the gastroprotective effects of ginger (*Zingiber officinale* Roscoe). Food Funct..

[6] Bartels E.M., Folmer V.N., Bliddal H., Altman R.D., Juhl C., Tarp S., Zhang W., Christensen R. (2015). Efficacy and safety of ginger in osteoarthritis patients: A meta-analysis of randomized placebo-controlled trials. Osteoarthr. Cartil..

[7] Wang J., Ke W., Bao R., Hu X., Chen F. (2017). Beneficial effects of ginger *Zingiber officinale* Roscoe on obesity and metabolic syndrome: A review. Ann. N. Y. Acad. Sci..

[8] Semwal R.B., Semwal D.K., Combrinck S., Viljoen A.M. (2015). Gingerols and shogaols: Important nutraceutical principles from ginger. Phytochemistry.

[9] Zick S.M., Djuric Z., Ruffin M.T., Litzinger A.J., Normolle D.P., Alrawi S., Feng M.R., Brenner D.E. (2008). Pharmacokinetics of 6-gingerol, 8-gingerol, 10-gingerol, and 6-shogaol and conjugate metabolites in healthy human subjects. Cancer Epidem. Biomar..

[10] Han Y., Li Y., Wang Y., Gao J., Xia L., Hong Y. (2016). Comparison of fresh, dried and stir-frying gingers in decoction with blood stasis syndrome in rats based on a GC-TOF/MS metabolomics approach. J. Pharm. Biomed..

[11] Mukkavilli R., Yang C., Tanwar R.S., Ghareeb A., Luthra L., Aneja R. (2017). Absorption, metabolic stability, and pharmacokinetics of ginger phytochemicals. Molecules.

[12] Bhattarai S., Tran V.H., Duke C.C. (2007). Stability of [6]-gingerol and [6]-shogaol in simulated gastric and intestinal fluids. J. Pharm. Biomed..

[13] Ozdal T., Sela D.A., Xiao J., Boyacioglu D., Chen F., Capanoglu E. (2016). The reciprocal interactions between polyphenols and gut microbiota and effects on bioaccessibility. Nutrients.

[14] Wang J., Wang P., Li D., Hu X., Chen F. (2020). Beneficial effects of ginger on prevention of obesity through modulation of gut microbiota in mice. Eur. J. Nutr..

[15] Minekus M., Alminger M., Alvito P., Ballance S., Bohn T., Bourlieu C., Carriere F., Boutrou R., Corredig M., Dupont D. (2014). A standardised static in vitro digestion method suitable for food—An international consensus. Food Funct..

[16] Mosele J.I., Macia A., Romero M.P., Motilua M.J., Rubio L. (2015). Application of in vitro gastrointestinal digestion and colonic fermentation models to pomegranate products (juice, pulp and peel extract) to study the stability and catabolism of phenolic compounds. J. Funct. Foods.

[17] Chen G.L., Chen S.G., Chen F., Xie Y.Q., Han M.D., Luo C.X., Zhao Y.Y., Gao Y.Q. (2016). Nutraceutical potential and antioxidant benefits of selected fruit seeds subjected to an in vitro digestion. J. Funct. Foods.

[18] Alqurashi R.M., Alarifi S.N., Walton G.E., Costabile A.F., Rowland I.R., Commane D.M. (2017). In vitro approaches to assess the effects of acai (*Euterpe oleracea*) digestion on polyphenol availability and the subsequent impact on the faecal microbiota. Food Chem..

[19] Condezo-Hoyos L., Mohanty I.P., Noratto G.D. (2014). Assessing non-digestible compounds in apple cultivars and their potential as modulators of obese faecal microbiota in vitro. Food Chem..

[20] Gayoso L., Claerbout A.-S., Isabel Calvo M., Yolanda Cavero R., Astiasaran I., Ansorena D. (2016). Bioaccessibility of rutin, caffeic acid and rosmarinic acid: Influence of the in vitro gastrointestinal digestion models. J. Funct. Foods.

[21] Valdes-Varela L., Ruas-Madiedo P., Gueimonde M. (2017). In vitro fermentation of different fructo-oligosaccharides by *Bifidobacterium* strains for the selection of synbiotic combinations. Int. J. Food Microbiol..

[22] Amato K.R., Yeoman C.J., Kent A., Righini N., Carbonero F., Estrada A., Gaskins H.R., Stumpf R.M., Yildirim S., Torralba M. (2013). Habitat degradation impacts black howler monkey (*Alouatta pigra*) gastrointestinal microbiomes. ISME J..

[23] Segata N., Izard J., Waldron L., Gevers D., Miropolsky L., Garrett W.S., Huttenhower C. (2011). Metagenomic biomarker discovery and explanation. Genome Biol..

[24] Langille M.G.I., Zaneveld J., Caporaso J.G., McDonald D., Knights D., Reyes J.A., Clemente J.C., Burkepile D.E., Thurber R.L.V., Knight R. (2013). Predictive functional profiling of microbial communities using 16S rRNA marker gene sequences. Nat. Biotechnol..

[25] Bustin S.A., Benes V., Garson J.A., Hellemans J., Huggett J., Kubista M., Mueller R., Nolan T., Pfaffl M.W., Shipley G.L. (2009). The MIQE guidelines: Minimum information for publication of quantitative real-time PCR experiments. Clin. Chem..

[26] Rahimlou M., Yari Z., Hekmatdoost A., Alavian S.M., Keshavarz S.A. (2016). Ginger supplementation in nonalcoholic fatty liver disease: A randomized, double-blind, placebo-controlled pilot study. Hepat. Mon..

[27] Wang J., Gao H., Ke D., Zuo G., Yang Y., Yamahara J., Li Y. (2013). Improvement of liquid fructose-induced adipose tissue insulin resistance by ginger treatment in rats is associated with suppression of adipose macrophage-related proinflammatory cytokines. Evid.-Based Compl. Alt..

[28] Kazeem M.I., Akanji M.A., Yakubu M.T., Ashafa A.O.T. (2013). Protective effect of free and bound polyphenol extracts from ginger (*Zingiber officinale* Roscoe) on the hepatic antioxidant and some carbohydrate metabolizing enzymes of streptozotocin-induced diabetic rats. Evid.-Based Compl. Alt..

[29] Dong J.L., Yu X., Dong L.E., Shen R.L. (2017). In vitro fermentation of oat *β*-glucan and hydrolysates by fecal microbiota and selected probiotic strains. J. Sci. Food Agric..

[30] Attri S., Sharma K., Raigond P., Goel G. (2018). Colonic fermentation of polyphenolics from Sea buckthorn (*Hippophae rhamnoides*) berries: Assessment of effects on microbial diversity by Principal Component Analysis. Food Res. Int..

[31] Wu T., Grootaert C., Pitart J., Vidovic N.K., Kamiloglu S., Possemiers S., Glibetic M., Smagghe G., Raes K., Van de Wiele T. (2018). Aronia (Aronia melanocarpa) Polyphenols Modulate the Microbial Community in a Simulator of the Human Intestinal Microbial Ecosystem (SHIME) and Decrease Secretion of Proinflammatory Markers in a Caco-2/endothelial Cell Coculture Model. Mol. Nutr. Food Res..

[32] Selma M.V., Espin J.C., Tomas-Barberan F.A. (2009). Interaction between phenolics and gut microbiota: Role in human health. J. Agric. Food Chem..

[33] Lee H.C., Jenner A.M., Low C.S., Lee Y.K. (2006). Effect of tea phenolics and their aromatic fecal bacterial metabolites on intestinal microbiota. Res. Microbiol..

[34] Scazzocchio B., Minghetti L., D’Archivio M. (2020). Interaction between Gut Microbiota and Curcumin: A New Key of Understanding for the Health Effects of Curcumin. Nutrients.

[35] Pluta R., Januszewski S., Ulamek-Koziol M. (2020). Mutual Two-Way Interactions of Curcumin and Gut Microbiota. Int. J. Mol. Sci..

[36] Sun Z.-Z., Li X.-Y., Wang S., Shen L., Ji H.-F. (2020). Bidirectional interactions between curcumin and gut microbiota in transgenic mice with Alzheimer’s disease. Appl. Microbiol. Biotechnol..

[37] Sanchez-Patan F., Cueva C., Monagas M., Walton G.E., Gibson M.G.R., Quintanilla-Lopez J.E., Lebron-Aguilar R., Martin-Alvarez P.J., Victoria Moreno-Arribas M., Bartolome B. (2012). In vitro fermentation of a red wine extract by human gut microbiota: Changes in microbial groups and formation of phenolic metabolites. J. Agric. Food Chem..

[38] Brown E.M., McDougall G.J., Stewart D., Pereira-Caro G., Gonzalez-Barrio R., Allsopp P., Magee P., Crozier A., Rowland I., Gill C.I.R. (2012). Persistence of anticancer activity in berry extracts after simulated gastrointestinal digestion and colonic fermentation. PLoS ONE.

[39] Hidalgo M., Oruna-Concha M.J., Kolida S., Walton G.E., Kallithraka S., Spencer J.P.E., Gibson G.R., de Pascual-Teresa S. (2012). Metabolism of anthocyanins by human gut microflora and their influence on gut bacterial growth. J. Agric. Food Chem..

[40] Hoffmann M., Messlik A., Kim S.C., Sartor R.B., Haller D. (2011). Impact of a probiotic *Enterococcus faecalis* in a gnotobiotic mouse model of experimental colitis. Mol. Nutr. Food Res..

[41] Liu B., Wang W., Zhu X., Sun X., Xiao J., Li D., Cui Y., Wang C., Shi Y. (2018). Response of gut microbiota to dietary fiber and metabolic interaction with SCFAs in piglets. Front. Microbiol..

[42] Topping D.L., Clifton P.M. (2001). Short-chain fatty acids and human colonic function: Roles of resistant starch and nonstarch polysaccharides. Physiol. Rev..

