# Supplementary material for: Assessing the Effects of Ginger Extract on Polyphenol Profiles and the Subsequent Impact on the Fecal Microbiota by Simulating Digestion and Fermentation In Vitro
Source: Nutrients. 2020 Oct 19;12(10):3194. doi: 10.3390/nu12103194 (PMC7650818; doi:10.3390/nu12103194)
Supplement: Supplementary file 1 [file nutrients-12-03194-s001.docx]

**Table S1.** Retention time, quantitation ion and calibration curves for major polyphenolic compounds of ginger.

| Compound | Retention Time (min) | Target Ion (m/z) | Qualitative Ion (m/z) | Calibration Curves | linear Range (μg/mL) | *R*^2^ |
| --- | --- | --- | --- | --- | --- | --- |
| 6-Gingerol | 1.23 | 277.20 | 177.02 | y = 904737x − 567239 | 1–50 | 0.9954 |
| 8-Gingerol | 1.40 | 305.04 | 177.02 | y = 378871x + 127312 | 1–50 | 0.9956 |
| 10-Gingerol | 1.63 | 333.05 | 177.02 | y = 16598x − 14254 | 1–50 | 0.9964 |
| 6-Shogaol | 1.46 | 277.20 | 137.10 | y = 225647x − 121539 | 1–50 | 0.9949 |
